# Effective treatment of SARS-CoV-2-infected rhesus macaques by attenuating inflammation

**DOI:** 10.1038/s41422-020-00414-4

**Published:** 2020-09-30

**Authors:** Shuaiyao Lu, Jingjing Zhao, Jiebin Dong, Hongqi Liu, Yinhua Zhu, Honggang Li, Liping Liu, Yun Yang, Shicheng Sun, Yifan Song, Yuan Zhao, Ruiping She, Tuoping Luo, Hongkui Deng, Xiaozhong Peng

**Affiliations:** 1 National Kunming High-level Biosafety Primate Research Center, Institute of Medical Biology, Chinese Academy of Medical Sciences and Peking Union Medical College, Kunming, Yunnan China; 2 State Key Laboratory of Medical Molecular Biology, Department of Molecular Biology and Biochemistry, Institute of Basic Medical Sciences, Medical Primate Research Center, Neuroscience Center, Chinese Academy of Medical Sciences, School of Basic Medicine Peking Union Medical College, Beijing, China; 3 School of Basic Medical Sciences, State Key Laboratory of Natural and Biomimetic Drugs, Peking University Health Science Center and the MOE Key Laboratory of Cell Proliferation and Differentiation, College of Life Sciences, Peking-Tsinghua Center for Life Sciences, Peking University, Beijing, 100191 China; 4 Peking-Tsinghua Center for Life Sciences, Academy for Advanced Interdisciplinary Studies, Peking University, 100871 Beijing, China; 5 Key Laboratory of Bioorganic Chemistry and Molecular Engineering, Ministry of Education and Beijing National Laboratory for Molecular Science, College of Chemistry and Molecular Engineering, Peking University, 100871 Beijing, China; 6 Laboratory of Animal Pathology and Public Health, Key Laboratory of Zoonosis of Ministry of Agriculture, College of Veterinary Medicine, China Agricultural University, Beijing, China

**Keywords:** Immunology, Cell biology

Dear Editor,

The COVID-19 pandemic caused by the SARS-CoV-2 virus has caused a significant public health crisis worldwide. Recent studies show that excessive inflammatory response is critical for SARS-CoV-2 pathogenesis and COVID-19 severity,^1^ which can lead to acute lung injury (ALI) and acute respiratory distress syndrome (ARDS).^2^ One major factor for acute inflammation in SARS-CoV-2 infection is the inflammatory macrophages, which has been considered important for the production of large amounts of proinflammatory cytokines.^3^ Autopsy identified an intense infiltration of macrophages in the lung tissues of fatal COVID-19 patients.^4^ Furthermore, single-cell RNA sequencing (scRNA-seq) showed a higher proportion of macrophages presenting in bronchoalveolar lavage fluid (BALF) of severe COVID-19 patients.^5^ Consistent with studies of SARS-CoV-2, infiltration and accumulation of macrophages in the lung were also found in other coronavirus diseases.^6^ Depletion of macrophages protected mice from lethal SARS-CoV infection, highlighting the important roles of macrophages in coronavirus-induced symptoms.^7^ Therefore, targeting macrophages to regulate hyperinflammation in SARS-CoV-2 infection could be an effective strategy to treat COVID-19 patients.

Recently, we developed a β-galactosidase (β-gal)-activated prodrug SSK1 that is able to effectively target macrophages, in which the expression of β-gal has been reported to be a physiological response to immune stimuli.^8^ We found that SSK1 treatment efficiently decreased macrophage numbers and their infiltration in lung injured mouse model, which accompanied with significant attenuation of inflammation.^9^ Basing on these results, we reasoned that SSK1 is a promising new drug that can be applied to target macrophages and treat excessive inflammation in COVID-19 patients. In this study, we investigated the therapeutic effects of SSK1 in SARS-CoV-2-infected nonhuman primate model.

Because SARS-CoV-2-induced pneumonia with hyperinflammation persisted and impaired lung function even after the peaks of SARS-CoV-2 virus infection,^10,11^ we studied the therapeutic effects of SSK1 on treating SARS-CoV-2-induced pneumonia at the late stage of infection using our previously established SARS-CoV-2-infected rhesus macaque model.^12^ In this model, the virus was almost cleared from 21 days post inoculation (dpi) (Supplementary information, Table S1). Nine rhesus macaques were divided into three groups: vehicle treatment (RM1–RM3), 0.5 mg/kg SSK1 treatment (RM4–RM6) and 2.0 mg/kg SSK1 treatment (RM7–RM9), each group contains one young, one adult, and one elderly monkey (Supplementary information, Table S2). Treatment was started from 22 dpi by intravenous injection for 7 consecutive days, and clinical data were collected for further analysis (Fig. 1a). Animals were euthanized for pathological analysis 5 days after the treatments stopped.

**Fig. 1:**
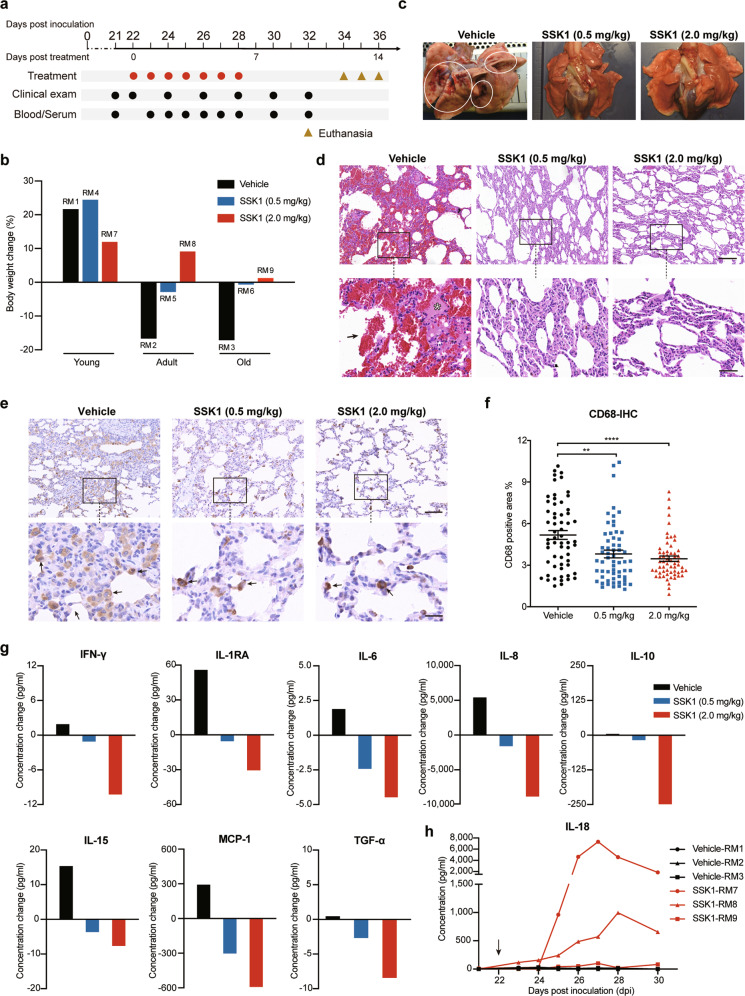
Improvement of COVID-19 pneumonia in rhesus macaques treated with SSK1. **a** Scheme of the experimental design of late-stage SSK1 intervention. Black dot, measurement of clinical signs and blood samples at the indicated time points. Red dot, treatment. Brown triangle, sample collections following euthanasia. **b** Body weight changes of SARS-CoV-2-infected monkeys with vehicle, low-dosage (0.5 mg/kg) or high-dosage (2.0 mg/kg) SSK1 treatment between 22 and 32 dpi. **c** Representative necropsy images of lungs showing that SSK1 treatment reduced the lesions of lungs from SARS-CoV-2-infected monkeys. Left, vehicle treatment (RM1); middle, SSK1 treatment, 0.5 mg/kg (RM4); right, SSK1 treatment, 2.0 mg/kg (RM7). White circle, lung lesions. **d** H&E staining images showing that SSK1 treatment improved the pneumonia of monkeys that were infected with SARS-CoV-2. Top, from left to right, representative H&E images of lungs of monkeys treated with vehicle (RM3), 0.5 mg/kg (RM6) or 2.0 mg/kg (RM9) SSK1; bottom, high magnification images of the boxed area in the top line. Arrow, hemorrhage. Asterisk, edema. Scale bars, top: 200 μm; bottom: 50 μm. **e** Immunohistochemistry (IHC) analysis for CD68 in lung tissues collected from SARS-CoV-2-infected monkeys treated with vehicle, 0.5 mg/kg or 2.0 mg/kg SSK1. Top, low-magnification images; bottom, high-magnification images of the boxed area in the top line. Scale bars, top: 100 μm; bottom: 25 μm. **f** Quantification of the IHC staining of CD68-positive cells in lung tissues after treatment. Analyzed in 20 random fields (0.75 mm^2^ per field) per animal, 3 animals per group. Each data point represents an independent field of view in the IHC slides. Data were analyzed with one-way ANOVA, all error bars represent SEM, **P < 0.01, ****P < 0.0001. **g** Concentration change of inflammatory cytokines in serum samples from old monkeys before (21 dpi) and after (28 dpi) SSK1 treatment. **h** Concentration measurement for IL-18 in serum samples from SARS-CoV-2-infected monkeys before and after SSK1 treatment (2.0 mg/kg). Arrow, starting point of administration.

We monitored the clinical signs throughout the experiment and found that SSK1 treatment effectively prevented body weight loss, whereas vehicle-treated adult and old monkeys showed obvious weight loss up to 17% (Fig. 1b). The body weight of all young monkeys kept increasing from 22 dpi, which was possibly because they were still at the developmental stage (Fig. 1b). We further analyzed lung injuries in the animals, and severe bright red lesions in the lungs were observed in gross examination in two of three vehicle-treated rhesus macaques (Fig. 1c; Supplementary information, Table S2), suggesting a continuous pneumonia. Importantly, among all six SSK1-treated monkeys, we only found one exhibiting slight lung lesions (Fig. 1c; Supplementary information, Table S2). Next, we investigated the therapeutic effects of SSK1 at the histopathological level. In the vehicle-treated group, hematoxylin and eosin (H&E) staining of lung sections showed a variable degree of thickened alveolar septum, edema and hemorrhage. These diseased phenotypes were greatly improved in the SSK1-treated groups (Fig. 1d; Supplementary information, Fig. S1, Table S2). The severity of histologic lesions was measured based on predetermined histology assessment criteria. Pulmonary histology assessment further showed a reduction in histologic lesions in SSK1-treated animals (Supplementary information, Table S2). Collectively, these results suggested that SSK1 improved pulmonary recovery from pneumonia of SARS-CoV-2 infection. Nonetheless, the pathological features in monkey models were not as severe as that in fatal COVID-19 patients, thus better animal models that mimic severe pathological state of COVID-19 patients are needed to evaluate SSK1 effect in the future study.

Next, we characterized the infiltration of macrophages by immunohistochemistry staining of the macrophage markers CD68 and CD163. A large number of CD68- or CD163-positive cells were found in the alveolar interstitium and a few within alveoli in vehicle-treated monkeys (Fig. 1e; Supplementary information, Fig. S2a), suggesting the infiltration and activation of macrophages after infection. SSK1 treatment significantly reduced macrophage infiltration, especially at high dosage (Fig. 1e, f; Supplementary information, Fig. S2a, b), which is consistent with our previous findings that SSK1 reduced macrophages in the ALI mouse model.^9^

To investigate the serum levels of various inflammatory cytokines that have been thought to play a key role in the progression of severe COVID-19 pneumonia, we compared the concentrations of 23 cytokines/chemokines in blood samples before (21 dpi) and after (28 dpi) SSK1 treatment. For the SSK1-treated old monkeys, inflammatory cytokines including IFN-γ, IL-1RA, IL-6, IL-8, IL-10, IL-15, TGF-α and MCP-1, were reduced at 28 dpi compared with 21 dpi, especially for those in the high-dosage group (2.0 mg/kg), suggesting attenuation of inflammation. In contrast, these cytokines were increased in vehicle-treated monkey (Fig. 1g). Anti-inflammatory treatments in aged individuals are of great importance in treating COVID-19 because this disease has higher mortality in senior populations who also have basal chronic inflammation.^13^ Interestingly, in all high-dosage SSK1-treated monkeys, we found an increased level of IL-18 (Fig. 1h), which has been found to be upregulated in COVID-19 patients during the period of rehabilitation.^10^ IL-18 has been reported as a protective cytokine against lung injury through reducing oxidative stress,^14^ which may play an important role in suppressing pulmonary fibrosis during the recovery stage. Accordingly, the upregulation of IL-18 during SSK1 treatment could be beneficial for modulating immune responses to antagonize COVID-19. Consistent with previously reported findings in SARS-CoV-2-infected monkey models,^11^ other cytokines such as TNF-α and IL-1β showed no significant change in our experiment. Collectively, our results suggested that SSK1 effectively regulated inflammatory cytokines in coordinating lung recovery in SARS-CoV-2-infected monkeys.

Finally, we also examined the safety of SSK1 treatment in the rhesus macaques. We did not observe any obvious side effects in rhesus macaques during the treatment of SSK1 (Supplementary information, Table S3). These findings were consistent with our recent toxicological assessments of SSK1 in mice, which showed that high-concentration (100 mg/kg) SSK1 treatment in mice had no apparent systemic toxicities. This safety profile was further supported by comparison to SSK1 effector gemcitabine, an approved clinical drug.^9^ Therefore, the in-vivo safety of SSK1 demonstrated in this and our recent studies provides a foundation for the potential of clinical applications in treating COVID-19 patients.

In summary, we demonstrated that the small molecule SSK1 effectively treated COVID-19 pneumonia by managing macrophage infiltration to attenuate inflammation. SSK1 efficiently mitigated clinical symptoms and pathologically reduced SARS-CoV-2-infected pneumonia (Fig. 1b–d), and there was a reduction in macrophage infiltration in the lungs of SSK1-treated animals (Fig. 1e, f). These results suggested the promising therapeutic potential of SSK1 in treating pneumonia and hyperinflammation in SARS-CoV-2 infection. We found that SSK1 decreased levels of cytokines in the peripheral blood of elderly monkeys (Fig. 1g). Previous studies have shown that patient age is an important prognostic factor in COVID-19 progression.^13^ To draw a definite conclusion of the relationship between SSK1 effects and age, more animals at different ages are needed to be tested in the future study. Considering that macrophages have also been shown to play a key role in antibody-dependent enhancement (ADE),^15^ transiently targeting macrophages by SSK1 may also have beneficial effects in the development of antibody-based therapies and vaccines. Therefore, targeting macrophages by SSK1 could be a promising strategy to control inflammation in COVID-19 treatment.

## Supplementary information

Supplementary Information

## References

[1] Moore JB, June CH (2020). Science.

[2] Tay MZ, Poh CM, Renia L, MacAry PA, Ng LFP (2020). Nat. Rev. Immunol..

[3] Merad M, Martin JC (2020). Nat. Rev. Immunol..

[4] Wang C (2020). EBioMedicine.

[5] Liao M (2020). Nat. Med..

[6] Page C (2012). J. Virol..

[7] Channappanavar R (2016). Cell Host Microbe.

[8] Hall BM (2017). Aging.

[9] Cai Y (2020). Cell Res..

[10] Wen W (2020). Cell Discov..

[11] Munster VJ (2020). Nature.

[12] Lu S (2020). Signal Transduct. Target. Ther..

[13] Zhou F (2020). Lancet.

[14] Nakatani-Okuda A (2005). Am. J. Physiol. Lung Cell. Mol. Physiol..

[15] Yip MS (2014). Virol. J..

