# Supplementary material for: Effective treatment of SARS-CoV-2-infected rhesus macaques by attenuating inflammation
Source: Cell Res. 2020 Sep 30;31(2):229–32. doi: 10.1038/s41422-020-00414-4 (PMC7525229; doi:10.1038/s41422-020-00414-4)
Supplement: Supplementary file 1 — Supplementary Information [file 41422_2020_414_MOESM1_ESM.pdf]

Supplementary information, Table S1. Viral RNA in nose, throat and anal swabs by RT-qPCR at different time points after infection.

Animal	Nose swabs			Throat swabs			Anus swabs		
	18 dpi	21 dpi	34 dpi	18 dpi	21 dpi	34 dpi	18 dpi	21 dpi	34 dpi
RM1	0.00	0.00	0.00	0.00	0.00	0.00	0.00	0.00	0.00
RM2	0.00	0.00	0.00	0.00	0.00	0.00	0.00	0.00	0.00
RM3	4.68	4.57	0.00	0.00	0.00	2.83*	0.00	0.00	0.00
RM4	0.00	0.00	0.00	0.00	0.00	0.00	0.00	0.00	0.00
RM5	0.00	0.00	0.00	0.00	0.00	0.00	0.00	0.00	0.00
RM6	0.00	0.00	0.00	0.00	0.00	0.00	0.00	0.00	0.00
RM7	0.00	2.94*	0.00	0.00	0.00	0.00	0.00	0.00	0.00
RM8	0.00	0.00	0.00	0.00	0.00	0.00	0.00	0.00	0.00
RM9	2.65*	3.14	0.00	2.65*	3.14	0.00	4.33	6.17	0.00

Viral load (log10 copies/ml), * means below the limit of detection.

Supplementary information, Table S2. Clinical and pathological observations in rhesus macaques infected with SARS-CoV-2 and treated with SSK1 or vehicle.

Treatment	Vehicle-DMSO			SSK1-0.5 mg/kg			SSK1-2.0 mg/kg		
**Animal**	RM1	RM2	RM3	RM4	RM5	RM6	RM7	RM8	RM9
**Age**	Young 1-year	Adult 5-year	Old 15-year	Young 1-year	Adult 5-year	Old 17-year	Young 1-year	Adult 5-year	Old 18-year
**Body weight change% (32 dpi vs 22 dpi)**	+ 22.22	- 17.24	- 17.72	+ 25.00	- 3.45	- 1.25	+ 12.50	+ 9.68	+ 1.82
**Necropsy**	Obvious bright red lesions in lung	No visible lesion in lung	Obvious bright red lesions in lung	No visible lesion in lung	Slight lesions in lung	No visible lesion in lung	A very small lesion in lung	No visible lesion in lung	A dark obsolete lesion in lung
**Pathological evaluation by H&E staining***									
Inflammatory cell infiltration	++	++	++	++	+	+	+	+	+
Hemorrhage	++	+	++	+	-	-	-	-	+
Thickened alveolar septum	++	+++	++	+	+	+	+	+	+
Edema	++	++	++	+	+	+	+	+	+

*Histology assessment criteria：We divided the characteristics of histologic lung lesions into four items, including inflammatory cell infiltration (including lymphocytes, neutrophils and monocyte-macrophages), hemorrhage, thickened alveolar septum and edema. Stained HE slides of lung lobes were analyzed and scored by pathologists based on the severity of each item (from - to ++++).

Supplementary information, Table S3. Evaluate the effects of SSK1 on liver and kidney function of rhesus macaques infected with SARS-CoV-2.

	Treatment	Animal	Before treatment	After treatment						
			21 dpi	23 dpi	24 dpi	25 dpi	26 dpi	27 dpi	28 dpi	30 dpi
AST (U/L)	Vehicle	RM1	52	52	30	33	36	43	53	39
		RM2	29	30	25	32	35	34	29	30
		RM3	31	29	32	35	34	42	27	25
	SSK1 0.5 mg/kg	RM4	91	69	50	39	40	37	22	38
		RM5	24	19	20	18	18	23	17	18
		RM6	74	44	35	40	31	46	28	28
	SSK1 2.0 mg/kg	RM7	61	56	38	32	59	56	44	46
		RM8	33	19	30	22	n.d.	28	24	26
		RM9	28	27	32	27	28	35	28	30
ALT (U/L)	Vehicle	RM1	21	39	29	25	26	29	28	26
		RM2	20	21	20	23	28	24	21	26
		RM3	13	16	14	17	19	21	17	18
	SSK1 0.5 mg/kg	RM4	26	44	46	39	31	33	19	28
		RM5	32	31	31	27	30	35	33	36
		RM6	74	65	50	46	39	47	34	41
	SSK1 2.0 mg/kg	RM7	32	37	39	29	37	35	34	32
		RM8	76	46	50	36	n.d.	35	34	33
		RM9	28	26	37	29	27	27	26	41

**UA (μmol/L)**	Vehicle	RM1	2	3	3	4	4	5	3	3
		RM2	5	2	7	2	3	3	2	3
		RM3	3	2	7	2	5	2	3	2
	SSK1 0.5 mg/kg	RM4	8	4	4	4	1	6	3	6
		RM5	5	4	7	3	5	3	2	2
		RM6	9	1	2	3	1	4	1	4
	SSK1 2.0 mg/kg	RM7	5	2	4	6	5	8	6	3
		RM8	13	4	3	2	n.d.	5	3	6
		RM9	10	0	2	5	6	3	1	5
**CRE-E (μmol/L)**	Vehicle	RM1	30	31	29	30	32	32	34	31
		RM2	70	56	52	58	64	61	74	72
		RM3	69	61	57	61	65	66	81	73
	SSK1 0.5 mg/kg	RM4	28	34	30	30	25	27	32	30
		RM5	55	41	38	31	40	38	48	41
		RM6	49	37	38	40	41	46	51	50
	SSK1 2.0 mg/kg	RM7	33	30	32	30	31	29	35	34
		RM8	55	45	50	42	n.d.	48	57	55
		RM9	58	46	57	50	52	54	63	50
**ALP (U/L)**	Vehicle	RM1	1398	1522	1229	1281	1360	1312	1283	1395
		RM2	152	128	116	141	171	154	124	173
		RM3	102	96	83	92	94	108	82	107
	SSK1 0.5 mg/kg	RM4	822	857	778	789	595	663	432	728
		RM5	124	94	86	66	74	75	67	89
		RM6	100	101	101	107	97	112	85	111

	SSK1 2.0 mg/kg	RM7	685	101	101	107	97	112	85	111
		RM8	427	280	365	269	n.d.	321	292	397
		RM9	233	192	250	203	212	226	188	192

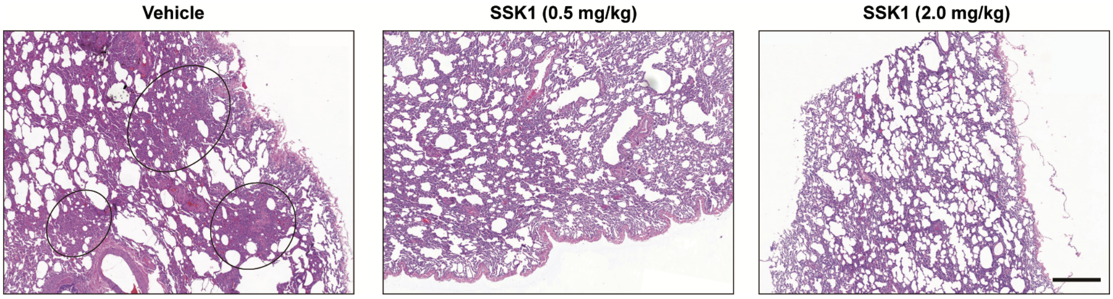

Supplementary information, Figure S1. Representative low magnification images of H&E staining shows that SSK1 treatment improved the pneumonia of monkeys infected with SARS-CoV-2**.** Circles show the area of interstitial pneumonia. Scale bar, 500 μm.

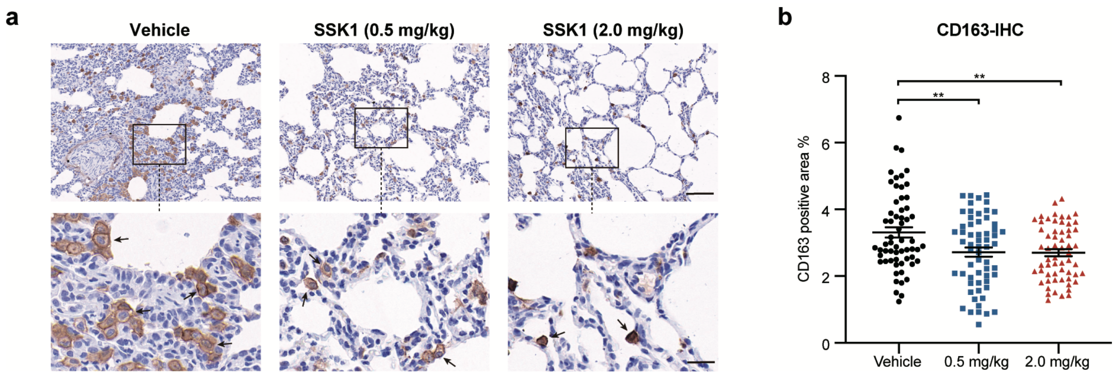

Supplementary information, Figure S2. a Immunohistochemistry (IHC) analysis for CD163 in lung tissues collected from SARS-CoV-2-infected monkeys treated with vehicle, 0.5 mg/kg or 2.0 mg/kg SSK1. Top, low magnification images; bottom, high magnification images of the boxed area in the top line. Scale bars, top: 100 μm; bottom: 25 μm. **b** Quantification of the immunohistochemistry staining of CD163-positive cells in lung tissues after treatment. Analyzed in 20 random fields (0.75 mm^2 per field) per animal, 3 animals per group. Each data point represents an independent field of view in the IHC slides. Data was analyzed with one-way ANOVA, all error bars represent SEM, ***P* < 0.01.

Materials and Methods

Ethics and biosafety statement

All animal experiments were performed in the animal biosafety level 4 (ABSL-4) facility of National Kunming High-level Biosafety Primate Research Center, Yunnan, China. All animal procedures were approved by the Institutional Animal Care and Use Committee of Institute of Medical Biology, Chinese Academy of Medical Science (Ethics number: DWSP202002 001). Rhesus macaques were monitored at least twice daily throughout the experiment. Commercial monkey chow, treats and fruit were provided daily by trained personnel.

Study design

To evaluate the therapeutic effect of SSK1 on the recovery period of SARS-CoV-2 infection, we used nine rhesus macaques of three different ages as reported before. All the animals were inoculated with total 4.75 ml of 10^6 pfu/ml SARS-CoV-2 intratracheally (4.00 ml), intranasally (0.50 ml) and on conjunctiva (0.25 ml). The nine rhesus macaques were divided into three groups according to the treatments, which included vehicle (DMSO), low dosage (0.5 mg/kg) and high dosage (2.0 mg/kg) of SSK1, respectively. Each group randomly selected one young, one adult and one old monkey. The final grouping is presented as following: the vehicle-treated group, RM1 (young), RM2 (adult) and RM3 (old); 0.5 mg/kg SSK1-treated group, RM4 (young), RM5 (adult) and RM6 (old); 2.0 mg/kg SSK1-treated group, RM7 (young), RM8 (adult) and RM9 (old).

As the experimental schedule showed in Figure 1a, we started the treatment on 22 dpi, and at this time the virus was almost undetectable. For each group, vehicle (DMSO), low dosage SSK1 (0.5 mg/kg) and high dosage SSK1 (2.0 mg/kg) were administrated intravenously (i.v.) once a day from 22 to 28 dpi. Animals were anesthetized and clinical exams were performed. We recorded the body weight, anal temperature on

each examination day and collected blood samples for hematology analysis as well as swabs form nasal, throat and rectal for virus quantitative detection. The monkeys were euthanized on 34, 35 and 36 dpi and recorded the pathological changes of lungs. Lung tissue samples were collected for further analyzed.

Drug treatment

100 mg/ml SSK1 dissolved in DMSO (Life Science, Cat# 1087C114) was mixed in 95% PBS (Gibco, Cat# 21-040-CVC), 5% Tween-80 (BioFroxx, Cat# 1716) and administrated intravenously (i.v.) at the dosage of 0.5 mg/kg or 2.0 mg/kg to monkeys at the indicated time point (the injection finished within 5-10 minutes). DMSO mixed in 95% PBS (Gibco, Cat# 21-040-CVC), 5% Tween-80 (BioFroxx, Cat# 1716) was intravenously (i.v.) injected as vehicle.

Virus amplification and identification

Viral stock of SARS-CoV-2 was obtained from the Center of Diseases Control, Guangdong Province China. Viruses were amplified on Vero-E6 cells and concentrated by ultrafilter system via 300 kDa module (Millipore). Amplified SARS-CoV-2 were confirmed via RT-PCR, sequencing and transmission electronic microscopy, and titrated via plaque assay (10^6 pfu/ml).

Cell culture

Vero-E6 cells were well cultured in high-glucose DMEM (Gibco, Cat# 11995500BT) supplemented with 10% fetal bovine serum (FBS, Gibco, Cat# 10099-141C) and 1% penicillin-streptomycin (Solarbio, Cat# P1400) in a humidified incubator at 37°C and 5% CO_2. Trypsin-EDTA (0.05%) phenol red (Gibco, Cat# 25200) was used for cell dissociation.

Hematology

The blood samples were collected from saphenous vein of hind limb of anaesthetized monkeys using 5 ml blood collection tube containing sodium citrate anti-coagulant for further analysis. For serum biochemical analysis, the blood was clotted for 30 min at 4°C and centrifugated at 3,000 × rpm for 15 min to obtain serum. The serum was used to analysis the alanine transaminase (ALT), aspartate transaminase (AST), alkaline phosphatase (ALP), uric acid (UA) and creatinine (CRE-E) by Chemistry Analyzer (Mindray, BS-350E).

Serum cytokine and chemokine analysis

The serum was collected for analysis of cytokine and chemokine. Concentrations of granulocyte colony-stimulating factor (G-CSF), granulocyte-macrophage colony-stimulating factor (GM-CSF), interferon (IFN)-γ, interleukin (IL)-1 receptor antagonist, IL-1β, IL-2, IL-4, IL-5, IL-6, IL-8, IL-10, IL-12/23 (p40), IL-13, IL-15, IL-17, IL-18, monocyte chemotactic protein (MCP)-1, macrophage inflammatory protein (MIP)-1α, MIP-1β, soluble CD40-ligand (sCD40L), transforming growth factor (TGF)-α, tumor necrosis factor (TNF)-α, vascular endothelial growth factor (VEGF) were determined using the Non-Human Primate Cytokine MILLIPLEX map 23-plex kit (Millipore, Cat# PRCYTOMAG-40K) according to the manufacturer's instructions.

Quantitative PCR

RNA extraction and quantitative real-time reverse transcription–polymerase chain reaction (qRT-PCR) were performed on nasal/throat/anal swabs collected from infected monkeys. The virus RNA was extracted from inactivated samples using Trizol LS Reagent (Invitrogen, Cat# 10296010) according to the Direct-zolTM RNA MiniPrep protocol (ZYMO RESEARCH CORP, US). 50 μl of DNase/RNase-Free Water was used to elute RNA. Real time RT-PCR was used to quantify viral genome in samples using TaqMan Fast Virus 1-Step Master Mix (ThermoFisher, Cat# 4444434) and purified viral RNA of SARS-CoV-2 as a standard curve, performed on CFX384 Touch

Real-Time PCR Detection System (Bio-Rad, US). Conditions for RT-PCR were used as follows: 25°C for 2 min, 50°C for 15 min, 95°C for 2 min, then 40 cycles at 95°C 5 sec and 58°C 31 sec. Primers and probe, specific for NP gene was synthesized according to sequences reported by Chinese Center for Disease Control and Prevention (CDC).

Target-2-F: GGGGAACTTCTCCTGCTAGAAT,

Target-2-R: CAGACATTTTGCTCTCAAGCTG,

Target-2-P: 5'-FAM-TTGCTGCTGCTTGACAGATT-TAMRA-3'.

In each run, standard dilutions of counted RNA standards were run in parallel, to calculate copy numbers in the samples.

Histopathology and immunohistochemistry

Necropsies of the rhesus macaques were performed according to a standard protocol. After the dissection, the major organs were grossly examined. The lung tissues were harvested, fixed in 10% neutral-buffered formalin solution, embedded in paraffin and 2 μm tissue sections were prepared. For Hematoxylin & Eosin (H&E) staining, slides were stained with Hematoxylin (Leica, Cat# 3801591) and Eosin (Leica, Cat# 3801594) (H&E) prior to microscopic pathologic analysis. Histopathological analysis of tissue slides was performed by pathologists blinded to the group assignment of the animals. To perform immunohistochemistry staining of macrophage, the anti-CD68 primary antibody (Abcam, Cat# ab213098) and anti-CD163 primary antibody (Servicebio, Cat# GB13340) was used. The corresponding second antibodies used were HRP conjugated Goat Anti-Mouse IgG (H+L) (Servicebio, Cat# GB23301) and HRP conjugated Goat Anti-Rabbit IgG (H+L) (Servicebio, Cat# G1215). The whole slide images were collected using Pannoramic DESK (3D HISTECH) and analyzed with Caseviewer C.V 2.3 and Image Pro plus 6.0.

Statistical analysis

For statistical analysis, *P* values were calculated by one-way ANOVA using GraphPad Prism 8 with default parameters. Error bars represent SEM and $P < 0.05$ was considered statistically significant (**$P < 0.01$, ****$P < 0.0001$).
